# Mitral Cells of the Olfactory Bulb Perform Metabolic Sensing and Are Disrupted by Obesity at the Level of the Kv1.3 Ion Channel

**DOI:** 10.1371/journal.pone.0024921

**Published:** 2011-09-22

**Authors:** Debra Ann Fadool, Kristal Tucker, Paola Pedarzani

**Affiliations:** 1 Program in Neuroscience, The Florida State University, Tallahassee, Florida, United States of America; 2 Department of Biological Sciences, The Florida State University, Tallahassee, Florida, United States of America; 3 Institute of Molecular Biophysics, The Florida State University, Tallahassee, Florida, United States of America; 4 Research Department of Neuroscience, Physiology and Pharmacology, University College London, London, United Kingdom; Sackler Medical School, Tel Aviv University, Israel

## Abstract

Sixty-five percent of Americans are over-weight. While the neuroendocrine controls of energy homeostasis are well known, how sensory systems respond to and are impacted by obesity is scantily understood. The main accepted function of the olfactory system is to provide an internal depiction of our external chemical environment, starting from the detection of chemosensory cues. We hypothesized that the system additionally functions to encode internal chemistry via the detection of chemicals that are important indicators of metabolic state. We here uncovered that the olfactory bulb (OB) subserves as an internal sensor of metabolism via insulin-induced modulation of the potassium channel Kv1.3. Using an adult slice preparation of the olfactory bulb, we found that evoked neural activity in Kv1.3-expressing mitral cells is enhanced following acute insulin application. Insulin mediated changes in mitral cell excitability are predominantly due to the modulation of Kv1.3 channels as evidenced by the lack of effect in slices from Kv1.3-null mice. Moreover, a selective Kv1.3 peptide blocker (ShK186) inhibits more than 80% of the outward current in parallel voltage-clamp studies, whereby insulin significantly decreases the peak current magnitude without altering the kinetics of inactivation or deactivation. Mice that were chronically administered insulin using intranasal delivery approaches exhibited either an elevation in basal firing frequency or fired a single cluster of action potentials. Following chronic administration of the hormone, mitral cells were inhibited by application of acute insulin rather than excited. Mice made obese through a diet of ∼32% fat exhibited prominent changes in mitral cell action potential shape and clustering behavior, whereby the subsequent response to acute insulin stimulation was either attenuated or completely absent. Our results implicate an inappropriate neural function of olfactory sensors following exposure to chronic levels of the hormone insulin (diabetes) or increased body weight (obesity).

## Introduction

It has been written that the olfactory system provides “an internal depiction of our external world” through the capture of odorant molecules in the main olfactory epithelium by several large families of G-protein coupled receptors. These receptors transduce the chemosignals into electrical signals that travel via topographically defined projections into the olfactory bulb [1]. We have uncovered that the mitral cells of the olfactory bulb, the first synaptic relay from the periphery to higher central targets such as the piriform cortex, function as internal chemical sensors of metabolic state by modulating a voltage-gated potassium channel predominantly expressed in these neurons [2]. Kv1.3, a mammalian homolog of the *Shaker* subfamily of potassium channels, carries a large proportion of the outward current in the mitral cell [3] and has multiple regulatory roles that are attributed to its structure and position as a central scaffold upon which tyrosine kinase signaling molecules form protein-protein interactions to modulate its function [4]–[6]. Gene-targeted deletion of Kv1.3 has revealed unusual non-conductive roles for this channel beyond those of traditional potassium channels, which are basically dampeners of excitability through timing of the interspike interval and shaping of the action potential, as well as controllers of the resting membrane potential [7]. Loss of function studies using whole-animal, targeted deletion of the Kv1.3 gene has demonstrated that the Kv1.3-null (-/-) mice have an enhanced olfactory ability in terms of threshold and discrimination of molecular features, supernumerary axonal projections to heterogeneous glomerular synaptic targets in the olfactory bulb, and increased expression of olfactory transduction machinery [8], [9]. Seemingly unrelated to the olfactory system, the Kv1.3-/- “Super-smeller” mice have metabolic alterations including an elevated energy expenditure and locomotor activity, irregular ingestive behaviors, resistance to diet- and genetic-induced obesity, and increased insulin sensitivity [8], [10]–[12]. In particular, when challenged with a moderately high-fat diet of 32% fat for 26 weeks, Kv1.3-/- mice fail to gain weight compared to their wild-type counterparts, and removal of the olfactory bulb via bilateral olfactory bulbectomy reverses their resistance to this diet-induced obesity (DIO) [8], [13].

Given our previous biophysical characterization of the Kv1.3 channel as a substrate for insulin phosphorylation and modulation [2], our goal was to determine the ability of the olfactory bulb to respond to changes in insulin blood chemistry driven by the physiological fluxes that would typically follow a meal (acute) or during metabolic disease or obese state (chronic). Using adult brain slices, we discovered that the duration of insulin stimulation drives changes in mitral cell action potential firing and shape. Mice develop a resistance to insulin modulation with the induction of an obese, diabetic state, which has an important impact on brain sensory function under varying states of energy utilization [14]. Our conclusion is that odorant code information that is inherent in spike firing frequency of mitral cells is intimately linked with metabolic state and concomitant endocrine function.

## Materials and Methods

### Ethics statement

All experiments described in this report were approved by the Florida State University Institutional Animal Care and Use Committee (IACUC) under protocol #9912 and were conducted in accordance with the American Veterinary Medicine Association (AVMA), the National Institutes of Health (NIH), and the UK Home Office regulations.

### Animal Care and Generation of Mutant Mouse Lines

All mice (strain C57BL6/J) were housed at the Florida State University vivarium in accordance with institutional requirements for animal care and were maintained on a standard 12/12 hour light/dark cycle. Kv1.3-null (-/-) mice were generated previously by deleting a large promoter region and the N-terminal third of the Kv1.3 coding sequence [12]. These mice were generously provided by Drs. Leonard Kaczmarek and Richard Flavell (Yale University, New Haven, CT).

### Diet-induced Obesity

For induction of diet-induced obesity (DIO), male mice were placed on a moderately high-fat diet (MHF)(Research Diets D12266B; 31.8% fat), using a matched chow diet (Purina 5001; 13.5% fat) as the control, as purchased from Research Diets (New Brunswick, NJ) and Lab Diets (Tallahassee, FL), respectively. For electrophysiology experiments, dams and experimental offspring were placed on the MHF-diet at P4 so that pups would receive the diet during postnatal development and throughout life until sacrifice for recording. All DIO mice were weighed daily or weekly and tail bleeds were sampled to probe for elevated blood chemistry prior to final use.

### Slice Electrophysiology

Adult mice (ages 35 to 85 days) were used for slice electrophysiology to facilitate insulin intranasal delivery and induction of DIO. Mice were anesthetized by inhalation of isoflurane (Aerrane; Baxter, Deerfield, IL), quickly decapitated, and then the olfactory bulbs (OBs) were exposed by removing the dorsal and lateral portions of the skull between the lambda suture and the cribriform plate. The brain was chilled in ice-cold oxygenated (95% O_2_/5%CO_2_) sucrose-modified artificial cerebral spinal fluid (sucrose ACSF; in mM: 83 NaCl, 26.2 NaHCO_3_, 1 NaH_2_PO_4_, 3.3 MgCl_2_, 0.5 CaCl_2_, 72 sucrose, 22 glucose; 315-325 mOsm, pH 7.3) for approximately 2 minutes prior to removal of the OBs for Vibratome sectioning (Vibratome Model 1000 or Leica VT1000S, Wetzlar, Germany). Coronal sections were made at a thickness of 275 µM and then allowed to recover in an interface chamber for 30 minutes at 33°C containing the oxygenated sucrose ACSF. Following incubation, sections were switched into oxygenated standard ACSF (in mM: 119 NaCl, 26.2 NaHCO_3_, 2.5 KCl, 1 NaH_2_PO_4_, 1.3 MgCl_2_, 2.5 CaCl_2_, 22 glucose; 305-310 mOsm, pH 7.3) and held at room temperature (rt) until use. OB slices were recorded in a continuously perfused (Ismatec; 2 ml/min), submerged-slice recording chamber (RC-26, Warner Instruments, Hamden, CT) using standard ACSF plus either synaptic blockers (5 µM NBQX and 25 µM APV; Ascent Scientific, Princeton, NJ) or various channel blockers (10 nM TTX, 50 pM ShK-186; Sigma Chemical or Bachem, Inc.) as deemed experimentally necessary in current- or voltage-clamp experiments, respectively. The intracellular pipette solution contained (in mM): 135 K gluconate, 10 KCl, 10 HEPES, 10 MgCl_2_, 2 Na ATP, 2 Na GTP; 280-285 mOsm, pH 7.3. Electrode position within the slice was visualized by an infrared, differential interference contrast microscope (Carl Zeiss, Axioskop 2 FS Plus) captured with an infrared camera (Dage MT1, CCD100). Electrodes were fabricated from borosilicate glass (Hilgenberg #1405002, Malsfeld, Germany) to a diameter of approximately 2 µm to yield a mean pipette resistance of 6.5±0.3 MΩ (n = 27). Positive pressure was retained while navigating through the OB laminae until a high resistance seal (R_e_ = 1.2 to 5.1 GΩ) was obtained on a positionally-identified mitral cell neuron in the slice. The whole-cell configuration was established by applying gentle suction to the lumen of the pipette while monitoring resistance. Each positionally-identified mitral cell was first sampled for adequate resting potential (<-55 mV) and proper series resistance (less than 40 MΩ) prior to initiating a series of current-clamp recordings. Typically cells were injected in 50–100 pA steps for 1000 ms to generate a family of evoked action potential trains and to determine the threshold to initiate a spike. Cells were then stimulated with a long, perithreshold current step (ranging from 5 to 50 pA) of 5000 ms every 10 s to acquire spike frequency data; prior and following 0.1 neµM (1 µg/mL) insulin (Roche, Florence, SC). Since mitral cell firing is intrinsically intermittent and is characterized by variable spike clusters, classical means of computing spike timing variability, such as peri-stimulus time histograms (PSTH), were less suitable for the behavior of these neurons and therefore alternative means of spike analyses were applied as described by Balu and Strowbridge (2004) [15].

### Data Acquisition and Statistical Analysis

Voltage-activated currents and current-evoked changes in membrane voltage were measured either using an Axopatch-1B integrating patch-clamp amplifier (Axon Instruments) or an EPC 10 (HEKA Elektronik). The analog signal was filtered at 2 kHz and minimally digitally sampled every 100 µs. Generation of voltage/current pulse protocols, data capture, and subsequent storage was either through pClamp9 (Axon) or through Patchmaster (HEKA). Data were analyzed in pClamp10 or in Pulsefit v8.6, in combination with the analysis packages Origin 6.0 (MicroCal Software), Quattro Pro 4X (Borland International), and Igor Pro 6.0.2 (Wavemetrics) with the NeuroMatics 2.02 plugin. Peak current magnitude and kinetics of inactivation and deactivation of the Kv1.3 current recorded in voltage-clamp were fit as previously described [8]. Spike analysis was performed using the event detection software, Neuromatics. Unless otherwise noted, the statistical design to compare electrophysiological parameters was a paired, within-cell metric so that a paired *t*-test could be applied at the 95% confidence level (α ≤0.05). Biophysical values were always expressed as the mean ± the standard error of the mean (s.e.m.).

### Insulin Intranasal Delivery

Insulin intranasal delivery (IND) was originally piloted for Sprague Dawley rats by Thorne et al., 2004 [16], which we modified for mice as fully described [14]. Briefly, for awake insulin IND, mice were hand-restrained, placed in a supine position, and given three, 10 µl drops of 5 µg/µl insulin or PBS vehicle, into both nares simultaneously. A meniscus of solution was formed and presented for inhalation by the awake mouse. Mice were given an extra 10 µl if the subject forcibly ejected or sneezed out solution. Mice were held supine for 5–10 sec after delivery to ensure all fluid was inhaled. It was previously shown that intranasally-delivered insulin-like growth factor reaches the OB with an efficiency of 0.11% [14]. Based upon those calculations and the equivalent molecular weight of insulin, it is estimated that 9 nM insulin is the effective dose of insulin reaching the OB. IND was repeated twice daily at 0700 and 1900 for eight days. On the morning of the ninth day, mice were given a final treatment at 0700, and sacrificed three hours later for slice electrophysiology.

## Results

Previous biophysical characterization of the cloned Kv1.3 channel has mapped the molecular targets of insulin modulation by tyrosine phosphorylation via mutagenesis approaches in the – and C-terminal aspects of the channel [2], [3]. Given the low levels of resting insulin in control or fat challenged Kv1.3-/- animals [13] and our finding that both the IR kinase and the Kv1.3 channel are resistant to phosphorylation following DIO in wild-type animals [14], lead us to further investigate the channel's function that could link metabolic state to excitability of neurons in a brain region expressing high levels of the channel [17]. We therefore sought to explore the modulation of the native Kv1.3 channel by insulin in the mitral cells of the olfactory bulb using an adult slice preparation. We hypothesized that the olfactory bulb could function as a sensor of metabolism by responding to levels of insulin, which would modulate the excitability of its output neurons, whose outward currents are largely contributed by Kv1.3 channels [3].

### Insulin Increases Mitral Cell Firing Frequency and Dampens Spike Adaptation

**Figure 1:**
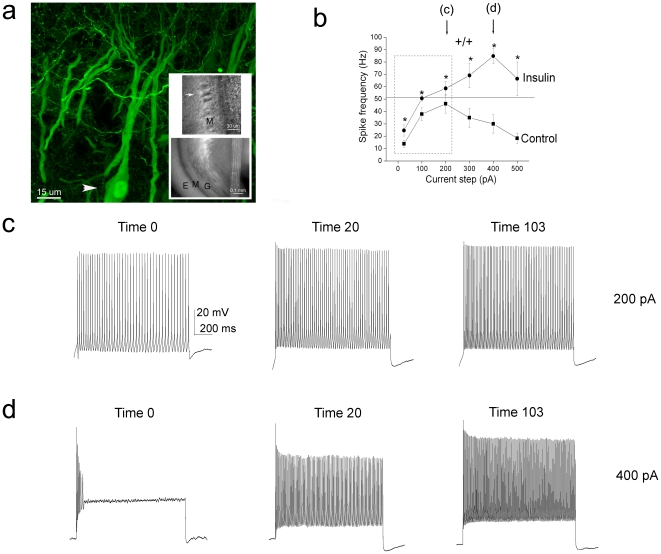
Acute insulin stimulation of mouse olfactory bulb mitral cells causes an increase in firing frequency and inhibits spike adaptation. (A) Laser confocal micrograph of an adult mitral cell (arrow head on soma) imaged from a YFP-thy1 mouse [19] as acquired in the mitral cell layer (bottom inset; bright field light micrograph). G =  granule cell layer, M =  mitral cell layer, E =  external plexiform layer. Top inset is a typical high power view of this lamina using infrared, differential interference contrast microscopy as captured with an infrared camera. Arrow  =  note the mitre shape of the neuron (hence its name) whose soma are highly concentrated in this lamina (M). (B) Line plot of the action potential spike frequency in response to various somatic current step injections as indicated. Data represent the mean ± the standard error of the mean (s.e.m.) of 8–10 recordings from neurons treated with control ACSF (Control) or with insulin (Insulin) for 20 minutes. *  =  Significantly different by paired *t*-test, α ≤ 0.05. Insets (lettered arrows) are demonstrated in subsequent panels. (C, D) Representative spike trains in a mitral cell that were elicited in response to a (C) 200 or (D) 400 pA current step of 1000 ms duration that were used to contribute to the line plot for insulin in panel A (20 minute time point; Time 20). Time 0 represents activity prior to insulin stimulation. Activity evoked after a long duration of insulin stimulation (103 minutes; Time 103) is also shown.

**Figure 2:**
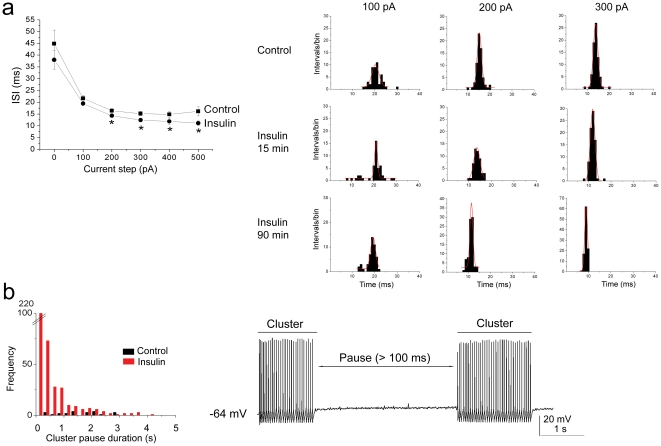
Insulin decreases the interspike interval (ISI) and cluster pause duration of mitral cell action potential activity. (A) (left) Line plot of the mean ISI in response to various somatic current injections as indicated, applied for a 1000 ms duration. Same notation, sample size, and statistical design as in Figure 1 except time point used for calculations was 15 minutes of insulin stimulation. (right) Histogram distributions of the interspike interval (ISI) for a representative neuron prior (Control) and following insulin stimulation taken at both the 15 and 90 minute time points (Insulin,15 min; Insulin, 90 min). Shown are three different current steps (100–300 pA). Plots were fit by Gaussian functions (solid red lines) to determine mean ISI as reported in panel A. *  =  Significantly different by blocked factorial ANOVA with Student Newman Kuels (*snk*) followup test, α ≤0.05. (B) (left) Frequency distribution plot of the pause duration between spike clusters for a neuron prior (Control, black bars) and following insulin stimulation (Insulin, red bars) taken at the 15 minute time point. (right) The neuron was stimulated using a 10 pA perithreshold current injection of 5000 ms duration, while recording near the natural resting potential of -64 mV. A pause was defined as a lack of spike activity for greater than 100 ms, while a cluster was defined as a minimum of 3 or more action potentials, but more typically 10 to 30.

A total of 172 cells from 81 mice were recorded across all combined electrophysiology experiments. Mitral cells were identified in the olfactory bulb slice based upon their pyramidal morphology when viewed under infrared differential contrast optics and position within the mitral cell layer (Figure 1A). Since there were no previous patch-clamp recordings reported for mitral cells of adult rodents, we used comparison to membrane properties described for postnatal animals [14], [18] as a secondary guide for positive identification, as well as YFP-thy1 mitral cell mice in a limited number of recordings while optimizing the viability of our slice preparation [19]. A sampling of the mean resting potential (RMP) of the adult mitral cells (61±1.8 mV; n = 12) and the mean input resistance (220±21.4 MΩ; n = 10) were in the same ranges reported in postnatal rats and mice. The mean firing frequency in response to graded step depolarizations of short duration ranging from 25 to 200 pA showed positive correlation in terms of number of spikes/s (Figure 1B) and a negative one in terms of the duration of the interspike interval (ISI) (Figure 2A). Application of insulin evoked a significant increase in firing frequency and a concomitant decrease in ISI at all ranges of current step amplitudes (Figure 1B and Figure 2A). Greater current step depolarizations (>200 pA) caused spike adaptation and a reduction in mean firing frequency in control recordings (Figure 1, see Time 0 in 1D). Most interestingly, insulin largely abolished spike frequency adaptation in response to the higher current injections (Figure 1D, Time 20, 103).

### Insulin Shortens Pause Duration Between Spike Clusters While Having Less Effect on Interspike Interval

**Figure 3:**
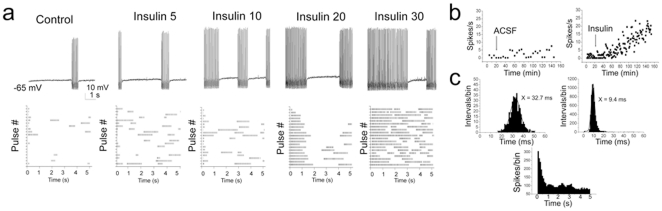
Insulin increases cluster length and decreases pause duration of perithreshold firing behavior in adult mitral cells. (A) Representative firing patterns (top) and the associated raster plots (bottom) generated in response to acute insulin stimulation over time in an adult mitral cell. Evoked activity was elicited using a 5 s long perithreshold current injection of 5 pA while recording near the natural resting potential of -65 mV. Numbers indicate time in minutes (i.e. Insulin 10, 10 minutes post bath application) compared to basal activity (Control). Each line of the raster plot represents action potential activity evoked during a 5000 ms pulse; complete raster is 20 consecutive pulses. (B) Example scatter plot of spike frequency of a neuron treated with ACSF (ACSF) versus that of one treated with insulin (Insulin) at the arrow. (C) (top) Example histogram distribution of the interspike interval (ISI) for a neuron prior (left) and following (right) insulin stimulation taken at Insulin 20. Plot was fitted by a Gaussian function (solid line) to determine mean ISI as reported in inset. (bottom) Example peri-stimulus spike distribution for the neuron in panel A at Insulin 20 to demonstrate change in first latency pattern of activity.

**Figure 4:**
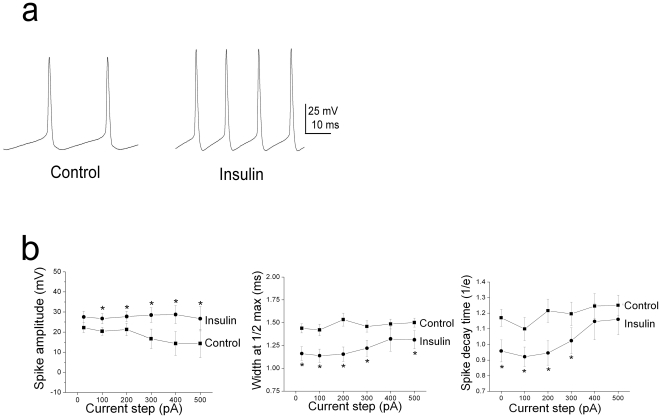
Insulin modulates action potential shape in adult mitral cells. (A) Example action potentials recorded in response to a 200 pA current injection prior (Control) and following (Insulin) bath application of the hormone. (B) Line graphs of the action potential amplitude (Spike amplitude), width (Width at ½ max), and falling phase (Spike decay time, 1/e) for a population of neurons recorded in ACSF (Control) and then following 20 minutes of bath application of insulin (Insulin) in response to various current steps as noted. *  =  significantly-different current step by paired *t*-test, α ≤0.05.

Mitral cells have been reported to undergo the firing of intermittent clusters of action potentials with a pause duration that is independent of the magnitude of the evoking current step and that persist in the presence of synaptic blockers [15]. To analyze this behavior, we defined a spike cluster as three or more action potentials preceded and followed by a pause of greater than 100 ms (Figure 2B). In addition to the ability of insulin to modulate ISI (calculated within a spike cluster or intracluster; Figure 2A), acute bath application of insulin for as little as 15 minutes, caused a significant shortening of the pause duration between spike clusters (calculated as intercluster) (Figure 2B). To further investigate the effect of insulin on this intrinsic firing activity observed in these neurons, mitral cells were stimulated with perithreshold current steps (5 to 50 pA) of long duration (5000 ms) while maintaining the cell near its RMP (Figure 3). Under these conditions, action potential clusters were clearly detectable and cells could continue firing for as long as three hours without significant changes in access resistance (mean value final R_a_ = 39.0±3.9 MΩ; n = 25). Significant changes in the spike frequency, intraburst frequency, latency to first spike, and the duration of the pause between spike clusters were first observed at the ten minute time point following application of insulin and became most prominent by visual inspection of the raster plot after twenty minutes of stimulation (Table 1, Figure 3A). Most noteworthy, the number of spikes/cluster and cluster length increased two-fold upon insulin stimulation, while the pause duration was significantly reduced by 40% (Table 1). The time course for the increase in the firing frequency of mitral cells in the adult slice preparation (Figure 3B) was consistent with our previous determinations of insulin-induced Kv1.3 current suppression in a primary cell culture preparation (voltage-clamp configuration), as well as insulin-dependent protein phosphorylation in native olfactory bulb and heterologous expression systems [2], [3]. A majority of the sampled cells (>85%) failed to return to baseline firing following 30 minutes to 2 h of washout with ACSF, also consistent with a persistent biochemical modification following insulin stimulation. Finally, analysis of the shape of the action potential, prior and following acute insulin application, was performed using graded-step depolarizing current injections of short duration and higher sampling frequency. Surprisingly, application of insulin for 20 minutes significantly reduced the width of the action potential (calculated at half-amplitude), increased the amplitude of the spike, and decreased the spike decay time (Figure 4, Table 1).

**Table 1:** Changes in biophysical properties in mitral cells following insulin application.

*Voltage clamp*	Control	Insulin
Peak transient current magnitude (pA)	3909.3±440.7 (5)	*2325.0±266.0
Peak sustained current magnitude (pA)	3273.3±319.6 (5)	*2195.0±305.2
Sustained/transient ratio (percent)	0.86±0.07 (5)	*0.74±0.03
τ_Inact_ (ms)	58.3±4.9 (4)	67.4±13.0
τ_Deact_ (ms)	24.7±4.6 (5)	34.7±9.7

*Current clamp – Evoked Firing Properties (25 pA)*		
Latency to first spike (ms)	1367.0±316.0 (9)	*158.0±91.3
Spike frequency (Hz)	13.8±2.6 (7)	*24.7±4.5
Intraburst frequency (Hz)	27.4±0.9 (9)	*33.4±2.7
ISI (ms)	44.8±5.8 (7)	38.0±4.0
Spikes/cluster	18.9±3.8 (9)	*42.4±6.4
Burst length (ms)	600.0±139.2 (9)	*1147.6±173.1
Pause duration (ms)	1054.6±138.4 (9)	*598.6±107.5

*Current clamp – Evoked Action Potential Properties (200 pA)*		
Peak reversal (mV)	21.3±3.1 (7)	*27.7±2.6
Width at half-maximum (ms)	1.5±0.07 (7)	*1.2±0.08
10–90% rise time (ms)	2.5±0.08 (7)	*1.7±0.09
1/e (ms)	1.2±0.08 (7)	*1.0±0.08

* =  Significantly-different mean values via paired *t*-test, α≤0.05. Insulin measurements represent the 20 minute time point following bath application of 0.1 µM (1 µg/mL) insulin. τ_Inact_  =  the inactivation time constant, τ_Deact_  =  the deactivation time constant, spike frequency  =  calculated throughout step depolarization, intraburst frequency  =  calculated during spike cluster, ISI  =  interspike interval within a spike cluster, action potential cluster  =  defined as three or more action potentials with less than 100 ms pause duration, pause duration  =  duration between clusters, 1/e  =  action potential decay time. Voltage-activated currents were measured using a Vh  =  -80 mV with a depolarizing Vc  =  +40 mV of 400 ms in duration and an interpulse interval of 30-45 s. In voltage-clamp recordings in which tetrodotoxin was not applied, a sustained/transient ratio was determined when spiking prevented subsequent kinetic analysis. In current-clamp recordings, firing properties were determined using a perithreshold current injection of 25 pA and 5000 ms duration. Action potential properties were determined using a 200 pA current injection of 1000 ms duration using a 50 µs sampling frequency.

### Mitral Cell Firing Is Modulated by Insulin at the Level of the Kv1.3 Channel

**Figure 5:**
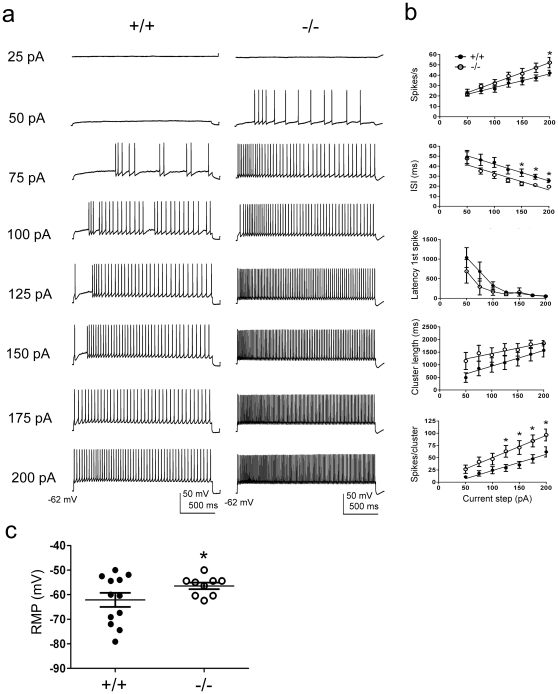
Spike firing properties in mitral cells compared across wild-type and Kv1.3-/- mice. (A) Representative action potential trains evoked by various current steps as indicated in a mitral cell recorded from a wild-type (+/+) and Kv1.3-null (-/-) mouse, respectively. Cells were recorded near the natural resting potential of -62 mV and evoked activity was elicited using a 2000 ms pulse duration. (B) Individual line plots of the mean spikes per second within a spike cluster (Spikes/s), the interspike interval (ISI), latency to first action potential (Latency 1^st^ spike), duration of the spike cluster (Cluster length), and number of spikes per cluster (Spikes/cluster) are shown in relationship to current step. Data represent mean ± s.e.m. values from a minimal of 20 recordings, acquired from 10–12 animals each, sampled from the two genotypes (+/+  =  closed circles, -/-  =  open circles). Data were fit by linear regression (lines), which were all significantly different by two-way analysis of covariance (ACNOVA) with respect to magnitude but not slope. *  =  significantly different by one-way ANOVA, α ≤0.05, Student Newman Kuels (*snk*) followup test. Note that Latency 1^st^ spike failed to be fit by linear regression, so only the latter statistical metric could be applied. (C) Scatter plot of the relationship of resting membrane potential (RMP) in mitral cells to genotype. Same symbols as in panel B; horizontal bar represents sampled mean, error bars represent s.e.m., and *  =  significantly different by Student's *t*-test, α≤0.05. See also Table 2.

**Figure 6:**
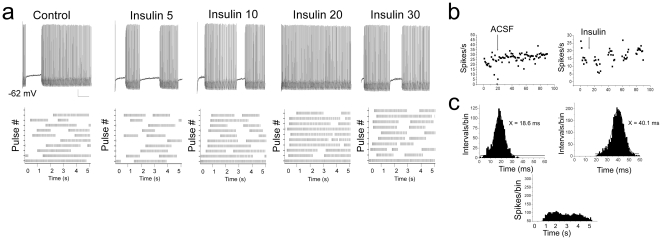
Insulin fails to significantly modulate mitral cell firing properties in Kv1.3-/- mice. Same as Figure 3 but for Kv1.3-/- mice as opposed to wildtype.

**Figure 7:**
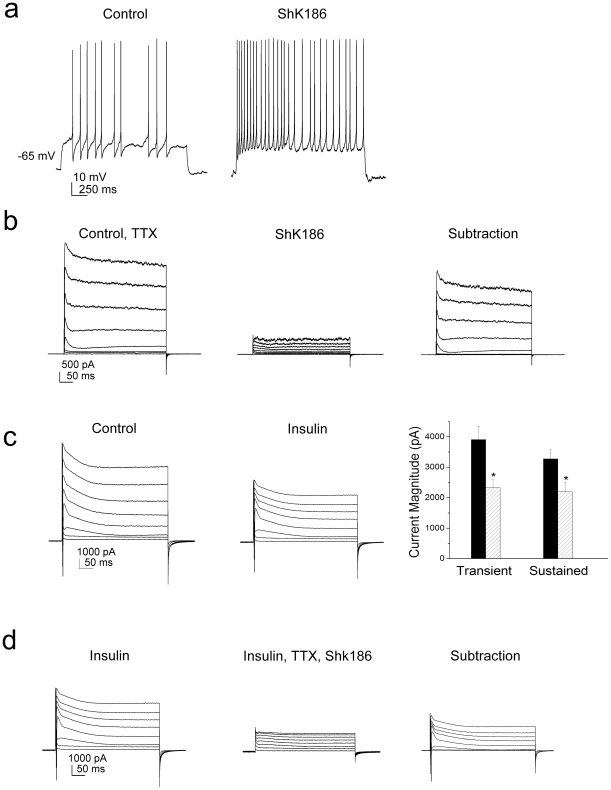
Insulin inhibits voltage-activated currents in adult mitral cells that are carried predominantly by Kv1.3 channels. (A) Representative current-clamp recording in a mitral cell prior (Control) and following a two minute bath application of the sea anemonie-derived channel peptide (ShK186). Evoked activity was elicited using a 1 s long current injection of 25 pA while recording near the natural resting potential of -65 mV. (B) Representative family of voltage-activated currents elicited in a mitral cell that was stepped in 10 mV depolarizing increments of 400 ms duration to a V_c_  =  +40 mV from a holding potential of -80 mV. Tetrodotoxin was added to control ACSF (Control, TTX) to eliminate unwanted sodium channel activity that would contribute to unwanted spiking. The subtraction trace represents the Kv1.3 contribution to the total mitral cell outward current. (C) (left) Same voltage paradigm as in panel B. The cell was first recorded in control ACSF (Control), followed by a 20 minute bath application of insulin (Insulin). (Right) Bar plot of the mean (± s.e.m.) peak transient or sustained outward current prior (black bar) and following insulin (stripped bar) stimulation. *  =  Significantly different by paired *t*-test, α≤0.05. (D) Same cell as in panel C, where TTX was then added to isolate outward currents and finally ShK186 was applied to uncover the Kv1.3 contribution that was modulated by insulin (Insulin, TTX, ShK186). The unidentified residual current is mediated by channels that are both insulin and ShK186 resistant. The subtraction trace represents the remaining Kv1.3 and Na channel contributions that were not modulated by insulin.

If the modulation of Kv1.3 was a predominant means of metabolic sensing for the olfactory bulb, then we hypothesized that the neurons should be insensitive to insulin in the absence of the channel. To test whether the changes in mitral cell firing properties induced by insulin were specifically mediated by Kv1.3 channels, we explored neuromodulation in slices prepared from adult Kv1.3-/- mice. We first characterized the basic biophysical properties of mitral cells in slices from adult Kv1.3-/- mice, since this has never been reported. Here we show that in comparison with wild-type mice, mitral cells lacking Kv1.3 respond to lower current injections (lower threshold to first spike), display a more depolarized RMP, an increased firing frequency, a decreased latency to the first spike, and a decreased pause duration between spike clusters (Figure 5). Basal biophysical values are compared across genotypes in Table 2. Using an identical perithreshold current injection paradigm as for wild-type neurons (Figure 3), we found that the firing behavior of mitral cells in slices from Kv1.3-/- mice was largely insensitive to the application of insulin (Figure 6). In addition to loss of function experiments via gene knock-out to define the selective involvement of Kv1.3 in sensing insulin, we also used pharmacological means. Using a selective blocker of Kv1.3 (ShK186), which binds the vestibule of the channel at low pM affinity [20], we confirmed that block of the Kv1.3 protein in mitral cells evoked an increase firing frequency as predicted (Figure 7A). To determine the proportion of Kv1.3 conductance modulated by insulin, we additionally performed voltage-clamp experiments. We first demonstrated that ShK186 blocked a large proportion of the outward voltage-activated currents in mitral cells, demonstrating that Kv1.3 is a main contributor to the total potassium current in these neurons (Figure 7B). In a separate set of experiments, application of insulin caused significant reduction of the outward voltage-activated current with no effect on the kinetics of activation or inactivation (Table 1, Figure 7C). There is a residual, unidentified current that is both insensitive to insulin and ShK186 (Figure 7D) at concentrations previously demonstrated to phosphorylate and suppress Kv1.3 currents or selectively block Kv1.3 over other *Shaker* family members [3], [20]. Also shown in this figure is the ShK186 sensitive, Kv1.3 current that remains following insulin modulation (Figure 7D, subtraction).

**Table 2:** Biophysical properties in mitral cells compared across genotype.

*Voltage clamp*	Wildtype	Kv1.3-/-
Peak transient current magnitude (pA)	3525±265 (50)	*3108±232 (27)
Peak sustained current magnitude (pA)	2792±223 (49)	2447±291 (27)
Sustained/transient ratio (percent)	0.80±0.01 (49)	0.79±0.02 (27)
τ_Inact_ (ms)	104±16 (22)	
τ_Deact_ (ms)	17±8 (23)	

*Resting Membrane Potential*		
RMP (mV)	-63.2±1.1 (40)	*-57.9±1.0 (27)

*Current clamp – Evoked Firing Properties (100 pA)*		
Latency to first spike (ms)	324.3±96.1 (8)	*171.0±94.6 (8)
Spike frequency (Hz)	14.8±3.1 (9)	21.6±6.2 (8)
Intraburst frequency (Hz)	26.7±3.1 (9)	32.2±3.5 (8)
ISI (ms)	43.8±5.0 (8)	*31.2±3.5(8)
Spikes/cluster	24.4±7.2 (8)	*47.2±11.7 (8)
Burst length (ms)	989.5±286.3 (8)	1385.3±286.7 (8)
Pause duration (ms)	802±233 (5)	*529±123 (7)

*Current clamp – Evoked Action Potential Properties (50 pA)*		
Amplitude (mV)	72.6±2.4 (7)	*59.6±4.6 (7)
Hyperpolarization (mV)	-15.9±1.3 (7)	*-12.4±1.5 (7)
Width at half-maximum (ms)	1.4±0.1 (7)	1.5±0.1 (7)
10–90% rise time (ms)	0.53±0.03 (7)	0.61±0.04 (7)
1/e (ms)	1.2±0.06 (7)	1.2±0.08 (7)

* =  Significantly-different mean values via a Student's *t*-test, α ≤0.05. τ_Inact_  =  the inactivation time constant, τ_Deact_  =  the deactivation time constant, RMP  =  resting membrane potential, spike frequency  =  calculated throughout step depolarization, intraburst frequency  =  calculated during spike cluster, ISI  =  interspike interval within a spike cluster, action potential cluster  =  defined as three or more action potentials with less than 100 ms pause duration, burst length  =  duration of the cluster, pause duration  =  duration between clusters, 1/e  =  action potential decay time. Voltage-activated currents were measured using a Vh  =  -80 mV with a depolarizing Vc  =  +40 mV of 400 ms in duration and an interpulse interval of 30–45 s. In voltage-clamp recordings in which tetrodotoxin was not applied, a sustained/transient ratio was determined when spiking prevented subsequent kinetic analysis. In current-clamp recordings, firing properties were determined using a 100 pA current injection of 4000 ms in duration. Action potential properties were determined using a 50 pA current injection of 1000 ms in duration with a 50 µs sampling frequency.

### Neuromodulation by Insulin Depends on Duration of Exposure and Metabolic State

**Figure 8:**
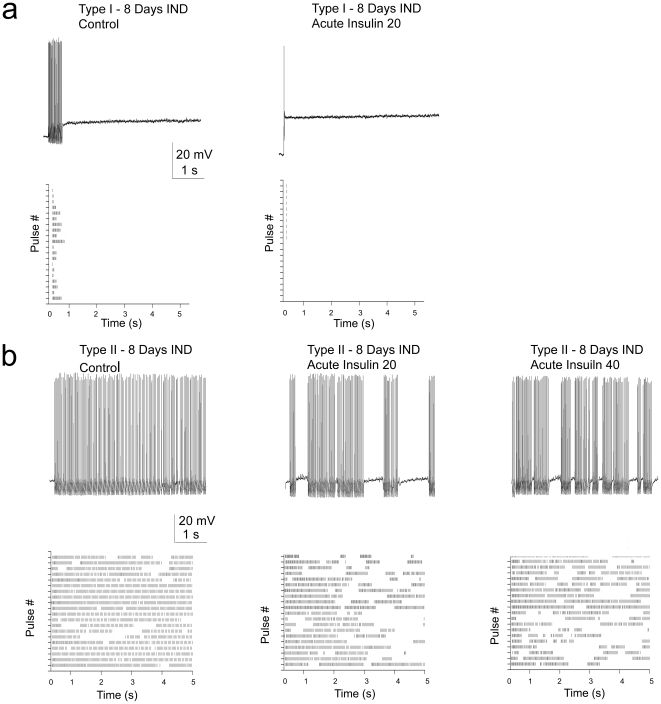
Insulin intranasal delivery (IND) changes basal firing behavior and leads to a reduced spike frequency following acute insulin stimulation. Representative basal action potential activity and the associated raster plot generated in response to an 8 day insulin IND (Control) and following subsequent, acute insulin stimulation over time (Insulin 20, Insulin 40). Two different patterns of basal action potential firing behavior were observed following insulin IND, (A) Type I and (B) Type II, as reported in the text. Time notations and raster plot generation as in Figure 3. Evoked activity was elicited using a 5 s long perithreshold current injection of 50 pA while recording near the natural resting potential of-63 mV. See also Table 3.

Long-term treatment with insulin may induce insulin resistance, a physiological state that is representative of chronic management of metabolic imbalance. We next tested the effect of chronic application of insulin by two methods, first by intranasal delivery of insulin over periods of days, and secondly by DIO that induces a prediabetic blood chemistry of elevated insulin. We hypothesized that chronic levels of insulin would modify biophysical properties of the mitral cells by altering Kv1.3 basal phosphorylation [14]. Adult mice of at least two months of age underwent intranasal delivery of insulin or PBS, twice daily for 8 days. This regimen does not modify peripheral levels of insulin or glucose, but it increases phosphorylation of IR kinase and Kv1.3 channel in the olfactory bulb and it enhances olfactory discrimination, among other behavioral phenotypes including anxiety and memory [14]. Following chronic insulin delivery, mitral cells displayed two distinct firing patterns (Figure 8) that were not observed with delivery of the PBS carrier alone. Sixty-four percent of recorded neurons (n = 11) fired only a single, short spike cluster with a short latency to first spike (Type I, Figure 8A), while 36% of recorded neurons (n = 4) had sustained firing with a very high initial firing frequency and short pauses between spike clusters (Type II, Figure 8B). Regardless of which firing pattern was observed following chronic intranasal insulin delivery, a subsequent acute exposure to insulin (20 minute application) now caused a pronounced lengthening of the pause duration, as opposed to increasing the excitability of the neuron, and in Type I mitral cells it additionally evoked a decrease in firing frequency (Table 3). The firing frequency in Type I mitral cells significantly decreased by 28%, while that of Type II neurons fell by 17% but did not reach statistical significance (see Table 3). In the latter class of adult mitral cells, the pause duration between spike clusters was significantly increased by 2 fold and the burst length was concomitantly decreased (Table 3).

**Table 3:** Changes in evoked firing properties in mitral cells following IND insulin application and diet-induced obesity.

*IND insulin application – 100 pA*	Basal	Insulin
**Type 1**		
Intraburst frequency (Hz)	41.3±12.4 (7)	*21.7±5.6
ISI (ms)	34.5±3 (7)	29.8±4
Spikes/cluster	2.6±1.1 (7)	*1.1±0.1
Burst length (ms)	79.8±21.4 (7)	none
Pause duration (ms)	none	none
**Type II**		
Intraburst frequency (Hz)	35.7±4.7 (4)	29.6±5
ISI (ms)	31.1±3.9 (4)	28.8±1.9
Spikes/cluster	37.1±6.4 (4)	29.8±0.4
Burst length (ms)	1319.5±382 (4)	*806.4±38.1
Pause duration (ms)	707.2±98.8 (4)	*1472.6±206.9

*Diet-induced obesity – 100 pA*		
**Spike Amplitude Changes**		
Intraburst frequency (Hz)	42.7±5.8 (6)	41.1±2.3
ISI (ms)	20.6±2.3 (6)	17.6±1.5
Spikes/cluster	25.5±12.3 (6)	12.0±3.6
Burst length (ms)	505.0±191 (6)	682.0±360.2
Pause duration (ms)	964.4±644 (6)	877.0±398.9
**Regular Amplitude**		
Intraburst frequency (Hz)	42.9±2.8 (10)	42.8±2.2
ISI (ms)	23.9±1.8 (10)	22.5±2.0
Spikes/cluster	22.1±3.7 (10)	*31.0±4.4
Burst length (ms)	568.8±93.5 (10)	*758.2±72.0
Pause duration (ms)	1848.7±201.3 (10)	*1408.1±152.7

* =  Significantly-different mean values via paired *t*-test, α ≤0.05. Insulin measurements represent the 20 minute time point following bath application of 0.1 µM (1 µg/mL) insulin. Intraburst frequency  =  calculated during spike cluster, ISI  =  interspike interval within a spike cluster, action potential cluster  =  defined as three or more action potentials with less than 100 ms pause duration, pause duration  =  duration between clusters. For these current-clamp recordings, firing properties were determined using a perithreshold current injection of 100 pA and 5000 ms duration.

**Figure 9:**
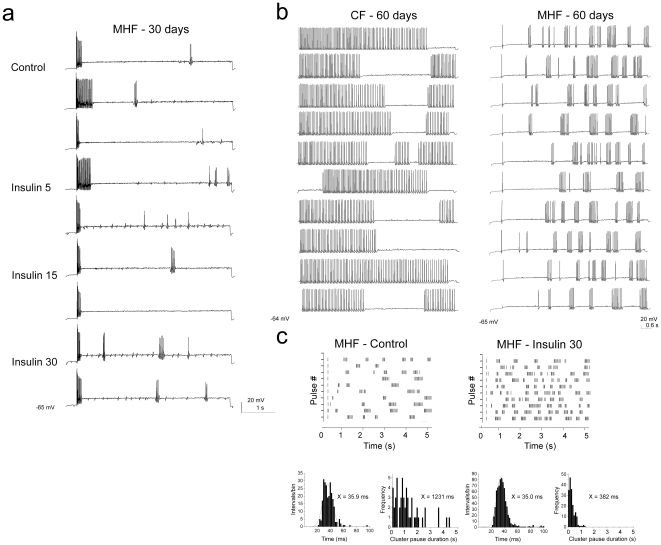
Diet-induced obesity affects action potential firing behavior in adult mitral cells and induces resistance to acute insulin stimulation. Following maintenance on a MHF diet ranging from 30 to 85 days, different patterns of action potential firing behavior were observed, two of which are illustrated in A and B: A) spike adaptation and partial spike amplitude firing and B) firing with shorter action potential clusters of long pause duration. In both A and B, evoked activity was elicited using a 5 s long perithreshold current injection while recording near the natural resting potential of -64 to -65 mV. (A) Representative action potential firing behavior in a mitral cell prior (Control) and following bath application of insulin for various time durations (5, 15, and 30 minutes) in a mouse that was maintained on a moderately high-fat (MHF) diet for 30 days. Note lack of increased spike firing frequency and numerous partial amplitude action potentials. (B) Representative action potential firing behavior in two different mitral cells that were acquired from sibling mice that were maintained for 60 days on either control purina chow (CF) or a MHF-diet (MHF). Note full action potential generation and differences in spikes/cluster, burst length, and pause duration. (C) (top) Raster plots of mitral cell firing activity generated from the cell in Panel B acquired from a MHF-diet maintained mouse. Spike firing behavior prior (MHF - Control) and following (MHF - Insulin 30) 30 minute bath application of insulin. Of the ten paired recordings performed that had regular amplitude action potentials (Panel B type), a few had weakly measurable increases in interspike, but not intraspike, firing frequency in response to insulin, but much lower than that of control fed animals (i.e. Figure 3A). (bottom) ISI histogram distribution and cluster pause distribution for Control and Insulin treated periods, respectively. Histogram plots were fitted by a Gaussian function (solid line) to determine the respective mean ISI as reported in insets or an algebraic mean was determined for the pause duration distributions as also reported in the insets. See full statistical analysis in Table 3.

The second way of chronically inducing insulin levels, in order to examine functional changes in mitral cell activity, was through DIO. In adult obese mice that had been challenged with a MHF-diet, three changes in action potential firing patterns were observed in comparison to control fed, aged-matched mice. Thirty percent of neurons (7 of 24) from fat challenged mice only fired a single action potential in response to a minimal current step of 200 pA (data not shown). Twenty-five percent (6 of 24) of recorded cells exhibited changes in spike amplitude as shown in Figure 9A. In these mitral cells, action potentials did not display full amplitude, there was marked spike adaptation, and the threshold to the first evoked spike was often two fold greater, even though the RMP did not significantly differ between control and MHF-fed animals (Obese  = -63.6±0.8 mV (n = 40) vs. Control -64.0±6.3 mV (n = 27); Student's *t*-test, α ≤ 0.05). The remaining 45% of sampled neurons (10 of 24) showed no changes in action potential amplitude, however, the basal burst length was shorter and the ISIs within spike clusters were shorter than those of control fed animals (see Table 2 vs. Table 3 (100 pA step); Figure 9B,C). Unlike mice for which insulin was chronically and centrally delivered intranasally, obese mice had elevations in both glucose and serum insulin levels (data not shown) and their firing patterns exhibited only slight responses to acute stimulation with insulin (Figure 9A–C; Table 3). Mitral cells displayed no response (Figure 9A) or an attenuated increase in spike frequency (Figure 9C) upon acute insulin stimulation compared with cells from control fed animals (Figure 3A), and had either no reduction or a blunted reduction in pause duration and only a 30% increase in burst length (Table 3). The results of the combined chronic experiments, in which insulin was either elevated by IND or DIO, demonstrate that the firing of these neurons in the olfactory bulb is highly state dependent; long-term exposure to the hormone insulin or a state of obesity can perturb modulation of intrinsic excitability, which in turn can change the relay of sensory information encoded by firing patterns.

## Discussion

This study reveals a new function of the olfactory bulb as an additional sensor of the body's metabolic state and internal chemistry, in addition to its well-recognized role to encode external chemosensory stimuli. We discovered a link between endocrine function, metabolism, and sensory neural activity that is molecularly centered around the Kv1.3 channel, predominantly expressed in mitral cells of the olfactory bulb. We found that the action potential firing properties of mitral cells are differentially modulated by acute and chronic levels of the hormone insulin. Moreover, the changes in action potential firing patterns, as well as the dampened response to insulin in mice made obese by a MHF-diet challenge, have a significant impact on the function of sensory systems in obese animals.

Our data predict that transient changes in insulin levels due to the ingestion of food would modulate mitral cell action potential frequency patterns to yield changes in electrical information relayed to higher processing centers. Early studies by Pager et al. [21] who monitored multi-unit electrical activity in the mitral cell layer in satiated compared with hungry rats, were the first to suggest that modulation of mitral cell activity may be driven by factors related to metabolic state. We now show that a strong modulator in the olfactory bulb is insulin. Acute exposure to insulin increases firing frequency and shortens the pause duration between spike clusters in mitral cells. It has been hypothesized [15] that the intrinsic membrane properties that drive intermittent spike clusters in the mitral cells are important for precisely timing spikes evoked by odor presentation to encode olfactory information. The spikes per cluster and length of the clusters are both enhanced by either gene-targeted deletion or pharmacological block of the Kv1.3 channel [8], [15]. The fact that insulin decreases the pause duration to enhance excitability, and the finding that Kv1.3-/- animals fail to significantly increase spike frequency upon insulin stimulation, are consistent with a central role of Kv1.3 channels in the modulatory action of insulin. Indeed, the evoked firing properties of wild-type neurons in response to insulin (Table 1) mirror those of untreated Kv1.3-/- mice (Table 2). In particular, the spikes/cluster, pause duration between spike clusters, and latency to first spike have significant similarities. Since activity patterns in mitral cells are thought to encode odor identity, it is interesting that insulin changes these patterns of activity in wild-type mice to a similar extent as that found in the Kv1.3-/- that have been previously characterized as “Super-smellers”. In particular, suppression or targeted-deletion of potassium conductances would be expected to principally shorten the latency to the first spike as we observed. Junek et al. (2010) [22] recently demonstrated that mitral cell spike latency was an excellent predictor of odorant identity and hypothesized that latency might be more important than frequency for coding. The slow onset of insulin modulation as well as its ability to dramatically curtail spike frequency adaptation, reveal, none-the-less, that the mitral cells have the capacity to double their firing frequency in a sustained manner upon acute hormone stimulation. Following chronic insulin exposure, the mitral cell appears to have a different set point in terms of firing properties, and a state dependency in respect to its response to additional acute insulin stimulation.

Neurons from animals with diet-induced obesity also displayed inherent physiological changes as reflected in their altered firing patterns both under basal conditions and in response to acute insulin stimulation. Diet-induced obesity, as well as genetic models of obesity, affect the expression and biophysical properties of channels and receptors in some tissues [23]–[25] and have no effect in others [26]. Our results provide a possible mechanism of how high-fat diet perturbs basal activity and insulin modulation of mitral cells, namely through the modulation of Kv1.3 activity. It would be very intriguing to reverse mice back to their original body weight to determine how persistent the changes in firing properties of mitral cells are.

The link between the olfactory system and energy balance should not be unexpected, given that perception of odorants drives food ingestion and selection [27]. It has been demonstrated that the nutritional status modulates both behavioral and immediate early gene expression in the olfactory bulb upon odor stimulation [28]. While our study focused upon the modulatory effect of insulin as one of the most important metabolic signals, orexins, leptin, and cholecystokinin have also been reported as regulatory molecules of olfactory behavior and odor-evoked and spontaneous activity in the olfactory epithelium [28]–[31]. The duration and target of the metabolic signal appears to differentially affect the olfactory-based physiological response. Savinger et al. [30] and Lacroix et al. [32] report a reduced electro-olfactogram response upon acute insulin stimulation at the epithelium. By contrast, chronic intranasal delivery of insulin increases olfactory discrimination but not odor threshold as determined by behavioral assessments [14], while increasing basal mitral cell firing rates (Figure 8). Subsequent opposite, or dampened modulation, by acute insulin stimulation following chronic changes in brain insulin (intranasal) or peripheral blood chemistry (DIO), demonstrate that there is a state dependency in the ability of the olfactory sensory system to detect metabolic changes. Lecoq et al. [33] have determined the relationship between capillary density and cellular metabolism, with fine spatial resolution in the olfactory bulb. Their measurements of tissue oxygen consumption during odor stimulation suggest that there is a great use of oxygen due to neuronal dendritic activation within the glomerulus. Parallel evidence in the modality of taste demonstrates that the metabolic status of the animal, in terms of level of GLP-1, glucagon, leptin, or endocannabinoids, for example, regulates sweet taste sensitivity [31], [34]–[36]. Yee et al. (2011) [37] recently reported the expression of glucose transporters, a sodium-glucose co-transporter, and two components of the ATP-gated K metabolic sensor in type 1 taste receptor 3-(T1r3) expressing neurons. Intriguingly, the chemoreception of fat has been proposed to involve the inhibition of a potassium delayed-rectifier channel, whereby an obesity prone strain of rats was found to be more sensitive in the detection of fatty acids and express higher channel densities [38]–[40].

Our data provide exciting lines of evidence for Kv1.3 channel as a potential sensor of metabolism within the OB. We have previously demonstrated that Kv1.3 is a target of insulin phosphorylation that exhibits resistance to phosphorylation following obesity [14]. Loss of Kv1.3 causes an increase in metabolism and a resistance to diet- and genetic-induced obesity [8], [10], [12] (Tucker et al., submitted). Moreover, in support of a central target for metabolism, we have recently demonstrated that Kv1.3-/- mice are no longer resistant to DIO and display decreased metabolism following bilateral olfactory bulbectomy [13]. A recent polymorphism in the human Kv1.3 gene that functionally elicits a gain in function has been associated with impaired glucose tolerance, lower insulin sensitivity, and impaired olfactory ability in male homozygous carriers [41], [42]. Kv1.3 channel, as a reported “diabetes risk allele” in humans, may represent an important candidate gene for therapeutic intervention [43]. Straub et al. (2011) [44] failed to alter glucose uptake by pharmacological inhibition of peripheral Kv1.3 channels, even though Kv1.3-/- mice have lower fasting glucose levels and lower blood glucose during a MHF-diet challenge [11], [12] (Tucker et al., submitted). Therefore, the Kv1.3 target for therapeutic intervention may be centrally located and our combined data support the OB as a potential locus. The fact that the action potential signaling output of the OB, as regulated by Kv1.3 channels, is modulated by the metabolic state of the animal, may provide an array of intriguing questions for future examination of the OB as an additional target for energy homeostasis and balance tied with odor perception and food selection.
